# Abnormal Pitch Perception Produced by Cochlear Implant Stimulation

**DOI:** 10.1371/journal.pone.0088662

**Published:** 2014-02-13

**Authors:** Fan-Gang Zeng, Qing Tang, Thomas Lu

**Affiliations:** Center for Hearing Research, Departments of Anatomy and Neurobiology, Biomedical Engineering, Cognitive Sciences, and Otolaryngology – Head and Neck Surgery, University of California Irvine, Irvine, California, United States of America; University of Chicago, United States of America

## Abstract

Contemporary cochlear implants with multiple electrode stimulation can produce good speech perception but poor music perception. Hindered by the lack of a gold standard to quantify electric pitch, relatively little is known about the nature and extent of the electric pitch abnormalities and their impact on cochlear implant performance. Here we overcame this obstacle by comparing acoustic and electric pitch perception in 3 unilateral cochlear-implant subjects who had functionally usable acoustic hearing throughout the audiometric frequency range in the non-implant ear. First, to establish a baseline, we measured and found slightly impaired pure tone frequency discrimination and nearly perfect melody recognition in all 3 subjects’ acoustic ear. Second, using pure tones in the acoustic ear to match electric pitch induced by an intra-cochlear electrode, we found that the frequency-electrode function was not only 1–2 octaves lower, but also 2 times more compressed in frequency range than the normal cochlear frequency-place function. Third, we derived frequency difference limens in electric pitch and found that the equivalent electric frequency discrimination was 24 times worse than normal-hearing controls. These 3 abnormalities are likely a result of a combination of broad electric field, distant intra-cochlear electrode placement, and non-uniform spiral ganglion cell distribution and survival, all of which are inherent to the electrode-nerve interface in contemporary cochlear implants. Previous studies emphasized on the “mean” shape of the frequency-electrode function, but the present study indicates that the large “variance” of this function, reflecting poor electric pitch discriminability, is the main factor limiting contemporary cochlear implant performance.

## Introduction

Pitch is a fundamental auditory percept that carries not only melodic information in music perception, but also speaker, prosodic or even lexical information in language processing. Pitch may be affected by sound pressure and waveform, but it primarily depends upon sound frequency [1]. Sound frequency is tonotopically organized as spatial location in the entire auditory pathway [2], [3], [4]. In the human cochlea, frequency from 20 Hz to 20,000 Hz is exponentially mapped to the 35-mm cochlear length, known as the Greenwood function [5]. Over this cochlear length, 3,500 inner hair cells are spatially arranged from apex to base and systematically tuned to different frequencies from low to high. As a result, a normal listener can detect approximately 1,400 steps of frequency differences for medium-loud sounds [6]. Although the detailed mechanisms are not clear, this tonotopic organization and sharp frequency selectivity are needed to support exquisite pitch perception. One example is the pitch of a harmonic sound, whose frequency spectrum (f) consists of integer multiples of a fundamental frequency (f = nF0, where n = 1, 2, 3, … and F0 = fundamental). A harmonic sound produces pitch at the fundamental, even if the fundamental itself is missing [7].

One design goal for a cochlear implant is to replicate this tonotopic organization and sharp frequency selectivity in a deafened ear. Contemporary multi-electrode cochlear implants have been successful in producing good speech perception in quiet, but fail to produce normal pitch perception [8]. In fact, most cochlear implant users cannot even recognize simple melodies [9], [10], [11], [12]. Several factors have been implicated in poor cochlear implant pitch perception. Vermeire et al. [13] studied 14 cochlear implant subjects who had unilateral implants but significant residual acoustic hearing in the non-implant ear. Using a high-resolution X-ray to identify electrode position in the cochlea and a pitch scaling method to identify the relationship of stimulation of a single electrode to pure tone frequency in the non-implant ear, they found a frequency-electrode function consistent with the Greenwood function for 9 of the 14 subjects. The remaining 5 subjects showed severe departure from the Greenwood function with either non-monotonic or flat functions. On the contrary, several similar studies found a frequency-electrode function that was consistently 1-2 octaves lower than the Greenwood function [14], [15], [16], [17], [18]. One reason for this inconsistency could be methodological differences [19]. The other reason was the lack of a gold standard in the non-implant ear, as reasonably good frequency selectivity above 500 Hz may be required for consistent pitch matches to electric stimulation [20]. Nevertheless, the misaligned frequency-electrode function has been assumed to be responsible for poor pitch perception in cochlear implants [13], [21], [22]. Hypothetically, this misalignment could be relatively easy to correct by individually changing the frequency to electrode map in the speech processor. The present study will directly test this hypothesis.

Relatively little is known about the discriminability of electric pitch. At best, the implant users could use pulse rates (up to several hundred Hz) to judge or produce music intervals [23], [24]. However, rate discrimination is an order of magnitude poorer than normal pure tone frequency discrimination [24], [25]. At the worst, 22 discriminable electrode steps [26] are 2 orders of magnitude less than 1400 discriminable frequency steps in acoustic hearing [6], [27]. The present study also aims to quantify electric pitch discriminability by comparing electric pitch to an established gold standard of pure tone pitch in acoustic hearing.

## Methods

### Subjects

**Figure 1:**
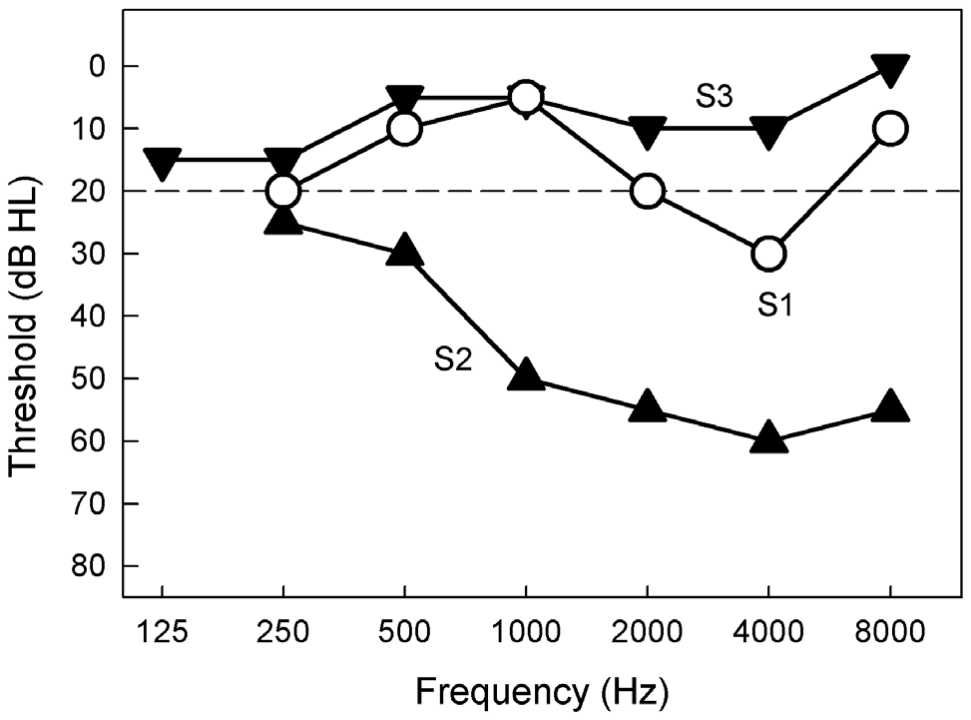
Audiograms for the non-implant ears. Hearing thresholds are plotted as a function of frequency for S1 (open circles), S2 (triangles), and S3 (inverted triangles). The dashed line indicates the conventional limit of normal hearing at 20 dB HL.

Three cochlear-implant subjects who had significant acoustic hearing in the non-implanted ear participated in the present study. All procedures in this study were approved by the University of California Irvine Institutional Review Board. Subjects gave written informed consent before data collection. Table 1 shows their demographic information. Figure 1A shows their audiograms. S1 was a musician and audio engineer and suddenly lost hearing on one side for unknown reasons. He received a HiRes90k device from Advanced Bionics Corporation (Valencia, CA) to treat his debilitating tinnitus [28]. In his clinical processor, the pulse rate was 2900 Hz per electrode and the pulse duration was 10.8 µs/phase. He had used the implant regularly and achieved 73% correct sentence recognition score that was typical of an implant user. In the non-implant ear, he had normal hearing (≤20 dB HL) at all tested frequencies except for 4000 Hz at which he had a mild loss of 30 dB HL.

**Table 1:** Subject information.

Subject	Age (years)	Duration of deaf (years)	Implant ear	Implant type	Duration of implant use (years)	Implant speech	Acoustic PTA (dB HL)	Acoustic melody
S1	46	1	Right	HiRes90K (1J)	1	HiRes-S (73%)	12 dB	100%
S2	59	12	Right	Combi40+ (medium)	5	HDCIS (97%)	45 dB	96%
S3	43	1	Left	PULSAR ci100 (medium)	1	HDCIS (43%)	7 dB	92%

Age is reported at the time of test. HiRes90k is a product of Advanced Bionics Corporation (Valencia, CA) and Combi 40+ and PULSAR ci100 are products of Med El (Innsbruck, Austria). Implant speech is specified as the type of speech processing and keyword percent correct for HINT sentences in quiet (all via direct connection to prevent acoustic leakage). PTA (pure tone average) is the average of thresholds in dB HL at 500, 1000 and 2000 Hz for the non-implant ear. Acoustic melody indicates percent correct recognition of 12 familiar melodies delivered to the non-implant ear.

S2 was a veteran and had fluctuating hearing loss most likely due to Meniere’s disease. He received a Med El implant (Innsbruck, Austria) and was the subject who had been thoroughly studied by Dorman et al. [18]. Except for his X-ray data, all data were collected independently in Irvine CA. In his clinical processor, the pulse rate was 1652 Hz per electrode and the pulse duration was 25.42 µs/phase. He used his implant on a daily basis and was considered a star user with 97% correct sentence recognition. In the non-implant ear, he had mild hearing loss (25 µ30 dB HL) below 1000 Hz and moderate hearing loss (50 µ60 dB) at 1000 Hz and above.

S3, who worked in finance, suddenly lost her hearing on one side and developed debilitating tinnitus on the same side for unknown reasons. She received a Med El implant to control her tinnitus. In her clinical processor, the pulse rate was 1587 Hz per electrode and the pulse duration was 25.42 µs/phase. She did not use the implant on a daily basis and scored 43% correct, which is a below-average sentence recognition score. She had normal hearing (≤15 dB HL) at all test frequencies.

### Stimuli

**Frequency discrimination.** Pure tones were used to measure frequency difference limens. The standard frequencies ranged from 125 to 8000 Hz in octave steps. The duration of each tone was 500 ms with 50-ms cosine-squared ramps. The level of each tone was presented at the individual’s most comfortable loudness. All pure tones were generated digitally and presented monaurally to the subject through a Sennheiser headphone (HDA200, Wedemark, Germany).

**Frequency-electrode function.** Pure tones used to match electric pitch were generated and presented in the same way as for measurement of frequency discrimination in the non-implant ear. All electric stimuli were generated and controlled by a research interface. For S1, electric stimuli were generated by the Bionic Ear Data Collection System software (BEDCS v1.17.208, 2006, Advanced Bionics Corporation, Valencia, California). The stimuli were 500-ms, charge-balanced biphasic pulse trains. The pulse duration was 107.8 µs/phase for the 100-Hz and 200-Hz pulse rates, and 53.9 µs/phase for the 2900-Hz pulse rate. For S2 and S3, electric stimuli were generated by a diagnostic interface box (CI STUDIO+ 2.02 software, Med El, Innsbruck, Austria). The stimuli were 500-ms, charge-balanced biphasic pulse trains. The pulse duration was 240.8 µs/phase for the 200-Hz pulse rate, and 24.2 µs/phase for the 1652-Hz and 2941-Hz pulse rates. Except for S1, pulse duration for relatively high rate (>1000 Hz) stimulation was similar to that used in the clinical processor. A much longer pulse duration was needed for low rate (100 or 200 Hz) stimulation to reach sufficient loudness in all subjects. This difference in pulse duration could affect perception between low- and high-rate electric stimulation, but would not affect pitch matching between acoustic and electric stimulation because pulse duration was held constant in each individual comparison. In all cases, the pulse trains were presented at the most comfortable loudness to each electrode in monopolar stimulation mode. Neither acoustic nor electric level was roved.

**Melody recognition.** Twelve familiar melodies were used to assess functional pitch performance [11]. The melodies consisted of 12 µ14 notes, with each note having the same rms level and 350-ms duration and a 150-ms silent interval between successive notes. The melodies were presented at the most comfortable loudness through either a Sennheiser headphone in the non-implant ear or a speech processor via direct electric connection in the implant ear.

### Procedures

**Intra-cochlear electrode position.** The same method as previously described [14], [29] was used to determine electrode position in the cochlea. Briefly, the modified Stenver’s view was used to identify the top of the semi-circular canal arch and the center of vestibule, producing a straight line connecting the top and the center; the round window position was then estimated as the intersection between this straight line and the electrode array. Each electrode position was determined relative to the round window. The estimation of the electrode position was corroborated by the surgeon’s visual report and subject’s perception of electric stimulation. For example, S3’s radiography showed that only 10 electrodes were inserted in the cochlea, which was consistent with the surgeon’s report and the subject’s dizzy or vestibular responses to stimulation of electrodes 11 and 12.

**Frequency discrimination.** A 2-alternative, forced-choice (2AFC) adaptive procedure [30] was used to estimate the difference limen. In each trial, the subject had to determine whether the first or the second sound had a higher frequency. Trial-by-trial feedback was provided. The initial frequency difference was large so that the subject could produce a correct response. Two consecutive correct responses led to a decrease in the frequency difference while any incorrect response led to an increase. The increasing or decreasing step size was logarithmically determined. A reversal was defined when an incorrect response occurred after consecutive correct responses or vice versa. The difference limen was the geometric mean of the 8 frequency values from 5 to 12 reversals, representing a 70.7% correct response level. Weber fraction, namely, the ratio of the difference limen over the standard frequency, was obtained as the measure of frequency discrimination.

**Frequency-electrode function.** For S1 and S2, a modified 2AFC double-staircase adaptive procedure [31], [32] was used to find pitch matches between electric stimuli in the implant ear and pure tones in the non-implant ear. For each electric stimulus, there were two randomly interleaved pure-tone sequences, one having an initial frequency that was deemed by the subject to evoke a clearly higher pitch than the electric stimulus and the other having a clearly lower pitch. In each trial, the subject first heard the electric stimulus, then the pure tone, and after that had to judge which sound had the higher pitch. In the first sequence, the pure tone frequency was decreased after the subject judged the pure tone to have the higher pitch than the electric stimulus two consecutive times but increased after the subject judged the pure tone to have the lower pitch any time. In the second sequence, the opposite decision rule was used. Different from the frequency discrimination procedure, the subject received no feedback regarding the correct response. Similar to the frequency discrimination procedure, reversal in the subject’s judgment direction was tracked in each sequence and the procedure terminated after at least 12 reversals were obtained for both sequences. The average of the last 8 reversals from the first sequence produced a pure tone frequency value that was judged higher in pitch 70.7% of the time compared to the electric stimulus, while that from the second sequence was judged higher in pitch 29.3% of the time. Mathematically, the average of these two values produced the pure tone frequency that was judged higher in pitch than the electric stimulus for 50% of the trials, or the point of subjective equality between electric and acoustic stimuli. Half of the difference between these two values estimated the difference limen in Hz for the electric stimulus, as it represented the distance between 50% and 70.7% points on the psychometric function.

For S3, the double-staircase adaptive procedure was not used due to time limitation. Instead, the subject was instructed to adjust the pure-tone frequency so that the pure tone was just higher in pitch than the electric stimulus, and then again so that it was just lower in pitch. The pure-tone frequency was adjusted on an unmarked scale from 20 to 20000 Hz. The point of subjective equality in pitch was the average of the just higher and just lower frequency values. The difference limen was half of the difference between these two values. Despite using a different method, S3 produced results similar to those for S1 and S2.

**Melody recognition.** A 12-alternative, forced-choice method was used to obtain percent correct scores using both the clinical map and the adjusted frequency-electrode function. To produce the adjusted frequency-electrode function, the cut-off frequencies for the bandpass filter in each channel were set to the just lower and just higher pitch matches for the corresponding electrode. The adjusted frequency-electrode function would correct electrode-pitch reversals (e.g., an apical electrode judged to have a higher pitch than an basal electrode), but would not eliminate redundant electrodes (i.e., two different electrodes judged to have overlapping pitches). Each melody was presented 3 times and the order of presentation was randomized. Melodies consist of well-known tunes such as “Twinkle Twinkle Little Star” and “Old Macdonald” [11].

## Results

### Frequency discrimination and melody recognition in the non-implant ear

**Figure 2:**
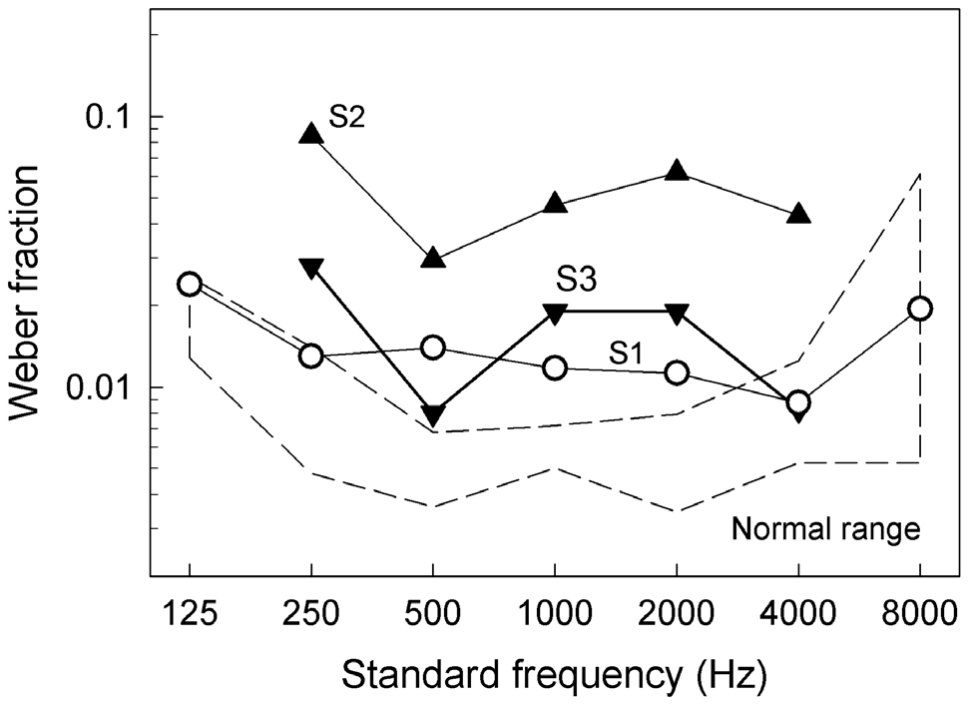
Frequency discrimination for the non-implant ears. The Weber fraction is plotted as a function of standard frequency for S1 (open circles), S2 (triangles), and S3 (inverted triangles). The normal range (mean±2 SDs) is shown as the dashed outlined box, which was obtained using a similar paradigm in a previous study [34].

Figure 2 shows Weber fraction values as a function of standard frequency. The mean of the Weber fraction over all standard frequencies for normal-hearing subjects was 0.013 (±0.010 SD). This normal control value was not significantly different from the mean of the Weber fraction for the non-implant ear of S1 (0.015±0.005 SD, p>0.05) or S3 (0.016±0.008 SD, p>0.05), but was significantly better than that of S2 (0.053±0.021 SD; 2-tailed, two-sample unequal variance t-test, p<0.05). S2’s Weber fraction value was similar to previous results obtained from subjects with cochlear damage [33]. Nevertheless, all 3 subjects produced nearly perfect melody recognition with their non-implant ears, namely, 100%, 96% and 92% for S1, S2 and S3 respectively (Table 1). These nearly normal results from the subjects’ non-implant ears validated their use as a “gold standard” for comparing electric pitch in their implant ears.

### Frequency-electrode function

**Figure 3:**
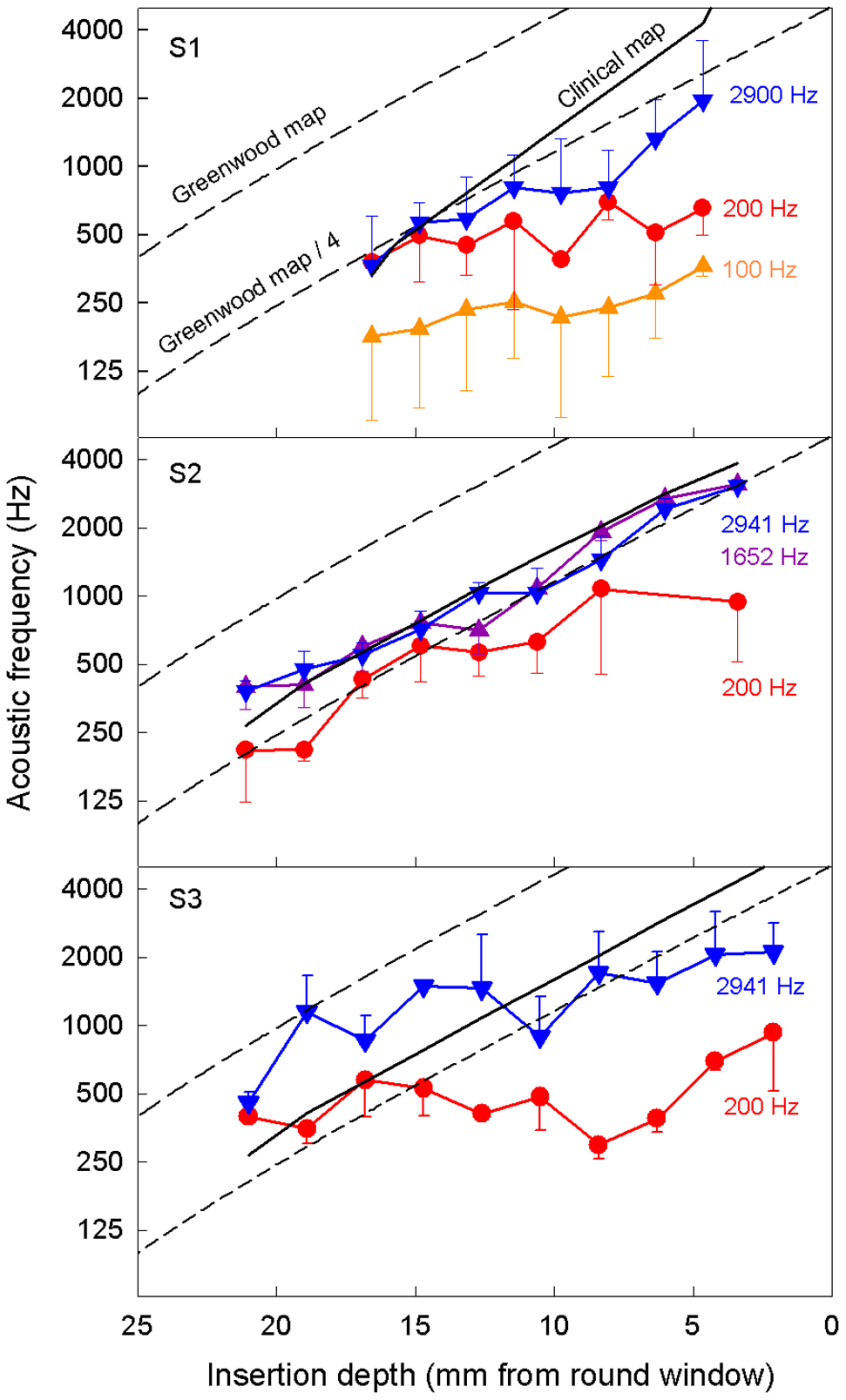
Frequency-electrode functions. The panel shows pure tone frequency matched to an electrode position for each of the 3 cochlear implant subjects with significant contralateral acoustic hearing. The x-axis is the electrode insertion depth from the round window. The y-axis is the pure-tone frequency matched to an electric stimulus delivered to a single electrode at a fixed stimulation rate (different symbols representing different rates). Error bars represent the frequency difference of two pure tones that were judged higher in pitch than the electric stimulus 50% and 70.7% of times (see text in the methods section). The upper dashed line represents the Greenwood function and the lower dashed line represents two octaves below the Greenwood function. The solid line represents the clinical frequency-electrode map used in the speech processor (Advanced Bionics for S1; Med El for S2 and S3).

Figure 3 shows pure tone frequencies matched to electric pitch as a function of electrode position. In each panel, the upper dashed line depicts the Greenwood function, and the lower dashed line depicts 2 octaves below the Greenwood function; the solid line depicts the clinical map that was used in the subject’s speech processor. The symbols represent the point of subjective equality obtained between acoustic and electric stimulation. The error bars represent the difference limen in Hz for electric pitch at its matched pure tone frequency, as defined in the Methods section and will be discussed in the next section.

There was great individual variability, but several trends were apparent in the data. First, there was a roughly monotonic trend between frequency and electrode position, with higher frequencies being matched to more basal electrodes. Second, there was a clear effect of pulse rate on the frequency-electrode function. The lower rates (≤200 Hz) produced lower pitches than the higher rates (>1600 Hz) at the same electrode position. Third, all frequency-electrode functions were much lower than the Greenwood function. S1 produced functions that were about 2 octaves below the Greenwood function for high-rate stimulation and even more for low-rate stimulation. The high-rate function also departed from the clinical map for basal electrodes (4 µ15 mm from round window). For S2, high-rate stimulation produced a function that was about 1 octave below the Greenwood function for the most apical electrode and about 2 octaves below for the most basal electrode. The measured function was reasonably close to the clinical map, with the former being slightly higher for apical electrodes and lower for basal electrodes. This high-rate result was similar to the result obtained by Dorman et al. from the same subject [18]. The additional 200-Hz rate stimulation for S2 produced a function that was at least 2 octaves lower than both the Greenwood function and the clinical map. For S3, both low and high rate stimulations produced a function that was mostly well below the Greenwood function, particularly for the basal electrodes. Finally, despite the 5-mm insertion difference between the Advanced Bionics (S1) and Med El (S2 and S3) implants, all 3 subjects matched their most apical electrodes to an acoustic pitch range between 200 and 400 Hz, reflecting possibly acclimation to the about 300 Hz lowest cutoff frequency in their speech processors (Table 1).

The slope of all measured frequency-electrode functions was calculated on a logarithmic frequency vs. linear distance scale. The mean slope of the measured functions was –0.075 (±0.047 SD; range  =  0.001 to –0.127), which was much shallower than the –0.154 slope for the Greenwood function. On average, the measured frequency-electrode function was 2 times more compressed in frequency range than the Greenwood function.

### Frequency discrimination

**Figure 4:**
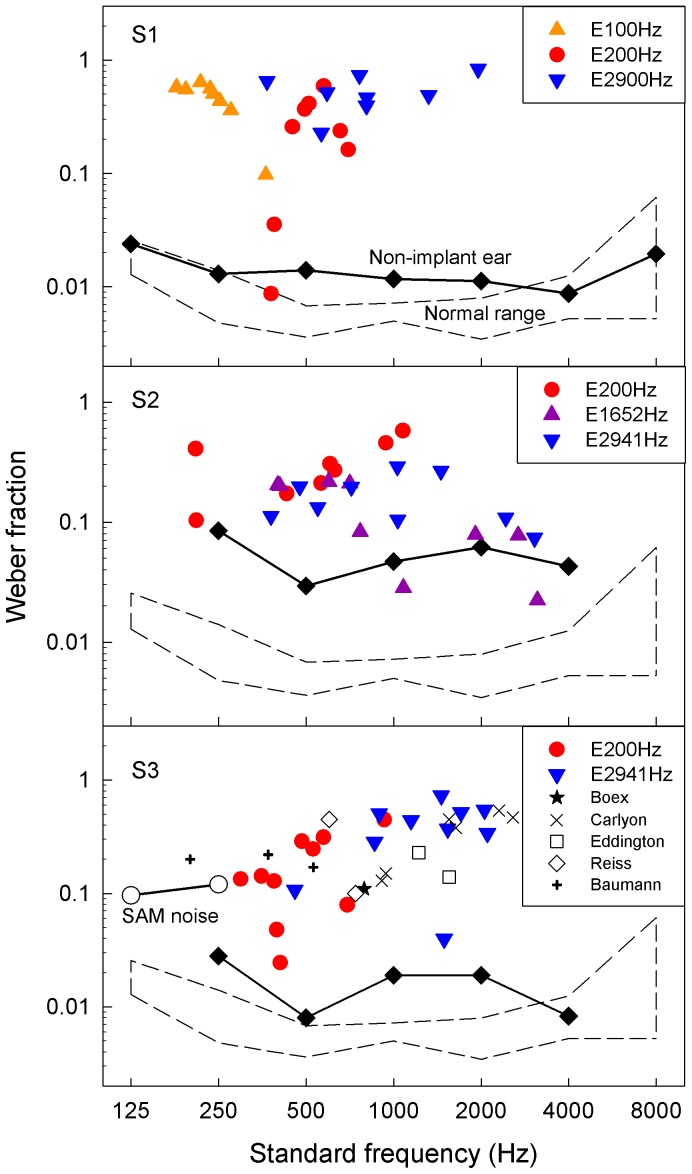
Weber fractions for acoustic and electric hearing. Normal-hearing subjects show relatively constant values (the dashed outlined box in each panel). The individual panels represent Weber fractions for 3 unilaterally implanted subjects: The filled diamonds represent the data for the non-implant ear while triangles and circles represent that for the implant ear. In panel S3 (bottom), comparable data from previously published other studies are also plotted: The two open circles represent Weber fractions for modulation frequency discrimination of sinusoidally-amplitude-modulated noise for a group of normal-hearing subjects [34]; the star represents the datum for a single implant subject from Fig. 4 of Boex et al. [14]; the six crosses represent the data for two subjects from Fig. 2 of Carlyon et al. [19]; the two open squares represent the data for a single subject from Fig. 22 of Eddington et al. [35]; the two open diamonds represent the data for two subjects from Fig. 1 of Reiss et al., in which the Weber fraction was estimated from the monotonic part of the psychometric function in S10 [21]; the three plus symbols represent the data from S1’s second estimates in Fig. 7 of Baumann and Nobbe [17].

Figure 4 shows the Weber fraction as a function of standard frequency from a number of experimental and control conditions. Compared with normal-hearing controls [34], the average Weber fraction for the 3 implant ears was 24 times worse (0.302 vs. 0.013, p<0.05). Compared with their non-implanted ears, it was 11 times worse (0.302 vs. 0.028). Individually, the Weber fraction in the implant ear was 29 times worse than that in the non-implant ear for S1 (0.422 vs. 0.015), 4 times for S2 (0.197 vs. 0.053), and 17 times for S3 (0.287 vs. 0.016; p<0.05 for all subjects). In fact, among all 70 experimental conditions from the 3 subjects involving electric stimulation, only 1 condition from S1 produced a borderline normal value (Weber fraction  =  0.009, the lowest red circle touching the dashed line). This lone electric stimulus was a 200-Hz pulse train delivered to the most apical electrode.

The bottom panel (labeled S3) shows 6 additional data sets for comparison. Like the present result, 5 previous studies involving 7 similar unilateral cochlear-implant subjects who had contralateral residual acoustic hearing [14], [17], [19], [21], [35] all produced abnormally large Weber fraction values. Because sinusoidally-amplitude-modulated noise is often suggested as an acoustic simulation of a cochlear implant [36], Weber fraction for modulation frequency discrimination of the same sinusoidally-amplitude-modulated noise was obtained in the same group of normal-hearing subjects as in the pure-tone frequency discrimination experiment. Indeed, the Weber fraction was abnormally large: 0.10 for 125-Hz and 0.12 for 250-Hz modulation frequency (open circles).

### Melody recognition

**Figure 5:**
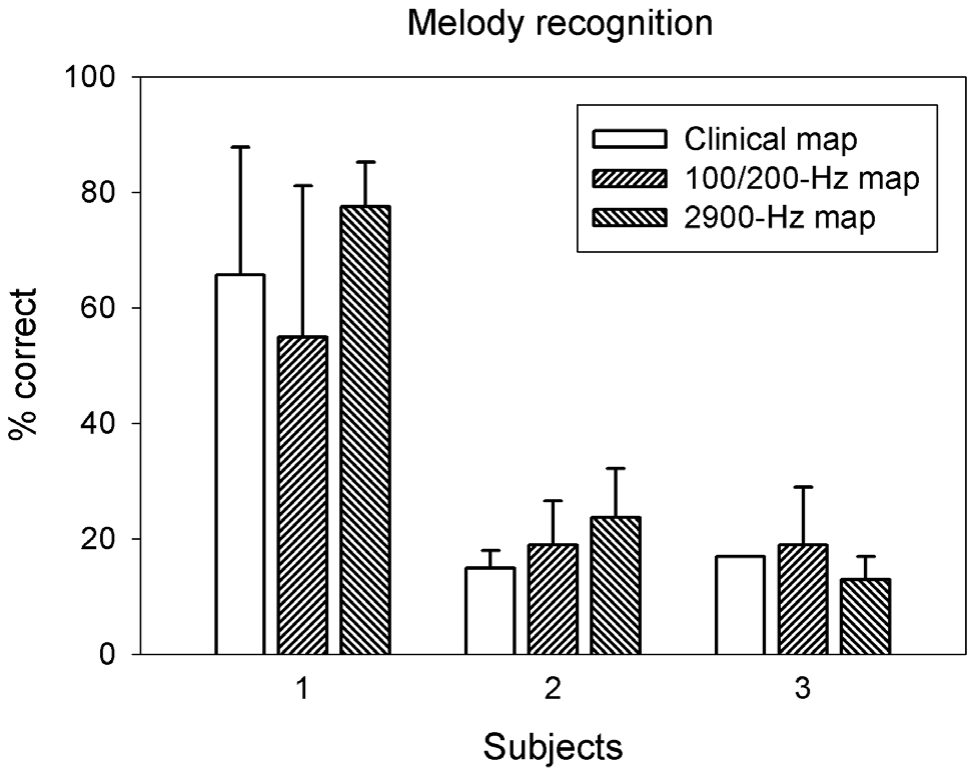
Melody recognition with the clinical map and the adjusted frequency-electrode functions. The results using the clinical map are shown as open bars, those using the low-rate adjusted function as upward-slanted bars, and those using the high-rate adjusted function as downward-slanted bars. Error bars represent one SD of the 3 repeated measurements.

Figure 5 shows melody recognition for the 3 subjects using either their clinical (open bars) or adjusted (slanted bars) maps. Despite more closely matched pitch between acoustic frequency and electrode position, none of the adjusted maps improved melody recognition over the standard clinical map. Because the band-pass filter cutoff frequencies were not systemically manipulated in the speech processor, it remained to be seen whether these cutoff frequencies could improve melody recognition. S1 had the highest melody recognition, perhaps as a result of his music training and experience. The other 2 subjects scored barely above chance. Note also that implant melody recognition was not related to implant speech recognition [37], as S1 produced modest, S2 nearly perfect, and S3 the lowest speech recognition (see Table 1).

## Discussion

### Poor electric pitch

Previous studies [13], [14], [15], [16], [17], [18], [19], [20] have emphasized on the mean values of the frequency-electrode function such as its shape, slope and range in relation to the hair cell or ganglion cell based cochlear frequency map [5], [22]. In the majority of cases, the obtained frequency-electrode function deviated from the normal cochlear frequency map. There is also evidence that both stimulation and experience can alter the mean frequency-electrode function in actual cochlear implant users. For example, the present study shows that low-rate stimulation produces shallower frequency-electrode function than high-rate stimulation. Reiss et al. [21] showed that the electrode pitch changed with time, “sometimes by as much as two octaves, during the first few years of implant use.” However, various studies including the present one attempting to adjust the frequency-electrode map did not fundamentally improve performance in actual cochlear implant users. The bottleneck must be somewhere else.

The present study suggests that the poor quality of electric pitch perception is mainly responsible for the limited cochlear implant performance. In fact, the 24 times larger Weber fraction for electric stimulation than acoustic stimulation likely represents the best-case scenario. The actual Weber fraction for electric stimulation could be worse than the present estimation because the cochlear implant user could have made lower or higher pitch judgment based on some other subjective quality (e.g., loudness, roughness, intermittency, timbre, or rattle rate). Previous studies using a multi-scaling approach came to a similar conclusion that electric stimulation, with either single- or dual-electrode stimulation, produces multi-dimensional percepts that are incompatible with the salient pitch evoked by a pure tone [38], [39], [40]. Also consistent with the present finding that adjusting the frequency-electrode function did not improve melody recognition, Spahr et al. [41] concluded that cochlear implant listeners “could likely identify an appropriate frequency-to-electrode map, but only in cases where the pitch strength of the electrically produced notes is very high.” Taken together, the previous and present results reinforce the notion that contemporary cochlear implants do not even remotely reproduce pitch sensation of a pure tone, let alone harmonic pitch [42].

### Implication for pitch models

Electric stimulation via contemporary intra-cochlear electrodes can produce a temporally precise neural firing pattern that is phase locked to stimulation at rates well above 1000 Hz [43], [44], but evokes a much broader than normal spatial excitation pattern [45], [46], [47]. The present results show that this temporally precise but spatially broad excitation pattern still produces a poor pitch percept. The poor electric pitch result is inconsistent with a large class of temporally-based pitch models, including the Licklider auto-correlation model [48] and its modern variations [49], [50], [51]. The present result is compatible with the suggestion that, in addition to precise temporal firing, a sharp spatial excitation that is located in a proper tonotopic place is required to produce a salient pitch percept like a pure tone or harmonics [25], [52].

### Ways to improve electric pitch quality

Although a cochlear spiral ganglion based map can potentially improve the frequency-electrode function [22], the present data suggest that adjustment of the frequency-electrode function is unlikely to improve cochlear implant pitch performance. The key is to develop a means of producing a spatially sharp excitation pattern, which can then be combined with a stimulation rate that is tonotopically appropriate. Two incremental approaches are worth exploring. First, current focusing with multiple electrode stimulation might be able to sharpen local excitation peak [53], but its effect on electric pitch quality has not been systematically explored. The present result suggests that it is important to identify proper stimulation rates for current focusing to improve electric pitch quality. Second, although implant subjects with residual acoustic hearing typically have hearing loss and poorer than normal frequency discrimination, the pitch quality from the residual acoustic hearing is still better than electric pitch (see Fig. 3). As a result, residual low-frequency acoustic hearing has been shown to enhance cochlear-implant melody recognition [54].

Two other approaches using novel stimulation might be considered as true disruptive technology. Different from diffuse electric stimulation, optical stimulation is focused and can produce a highly selective spatial excitation pattern [55]. Still using electric stimulation but with a penetrating electrode array, Middlebrooks et al. [56] found that intra-neural stimulation can also produce a highly selective spatial excitation pattern similar to that produced acoustically by pure tones. Moreover, the penetrating electrodes can access those low-frequency nerve fibers that reside in the middle of the auditory nerve trunk and are not normally accessible to traditional intra-cochlear electric stimulation [57]. Although these novel approaches are likely years away from human trials, they offer the hope of reproducing a salient pure tone sensation, and possibly normal harmonic and music perception in future users of auditory prostheses.

## Conclusions

The present study measured frequency-electrode functions and evaluated their effects on melody recognition for 3 unilateral cochlear implant subjects who had significant acoustic hearing over the entire audiometric frequency range in the non-implant ear. Compared with the Greenwood function, the averaged frequency-electrode function was lower in pitch and compressed in frequency range, particularly at low stimulation rates. Adjusted frequency-electrode functions did not improve cochlear implant melody recognition. The derived Weber fraction for electric pitch was 24 times worse than that for acoustic pitch, indicating that the large variance in electric pitch is the main cause for poor cochlear implant melody recognition.
